# Wnt Signaling and Biological Therapy in Rheumatoid Arthritis and Spondyloarthritis

**DOI:** 10.3390/ijms20225552

**Published:** 2019-11-07

**Authors:** Daniela Cici, Addolorata Corrado, Cinzia Rotondo, Francesco P. Cantatore

**Affiliations:** Rheumatology Clinic, Department of Medical and Surgical Sciences, University of Foggia, 71121 Foggia, Italy

**Keywords:** Wnt signaling, Dkk-1, biologics, rheumatoid arthritis, ankylosing spondylitis, axial spondyloarthritis, bone homeostasis

## Abstract

The Wnt signaling pathway plays a key role in several biological processes, such as cellular proliferation and tissue regeneration, and its dysregulation is involved in the pathogenesis of many autoimmune diseases. Several evidences support its role especially in bone complications of rheumatic diseases. In Rheumatoid Arthritis (RA), the Wnt signaling is implicated in systemic and localized bone loss, while available data of its role in Spondyloarthritis (SpA) are conflicting. In the last few decades, the quality of life of rheumatic patients has been dramatically improved by biological therapy, targeting cytokines involved in the pathogenesis of these diseases like tumor necrosis factor (TNF)α, interleukin (IL)-1, IL-6, IL-17. In this review, we reviewed the role of Wnt signaling in RA and SpA, focusing on the effect of biological therapy on this pathway and its possible clinical implications.

## 1. Introduction

Wnt signaling is a key pathway involved in several biological processes, such as cellular proliferation, cell migration, tissue homeostasis and regeneration, embryonic development and stem cell maintenance. Furthermore, the dysregulation of Wnt signaling is believed to be involved in the pathogenesis of cancer, vascular disorders and autoimmune diseases [1]. 

**Figure 1:**
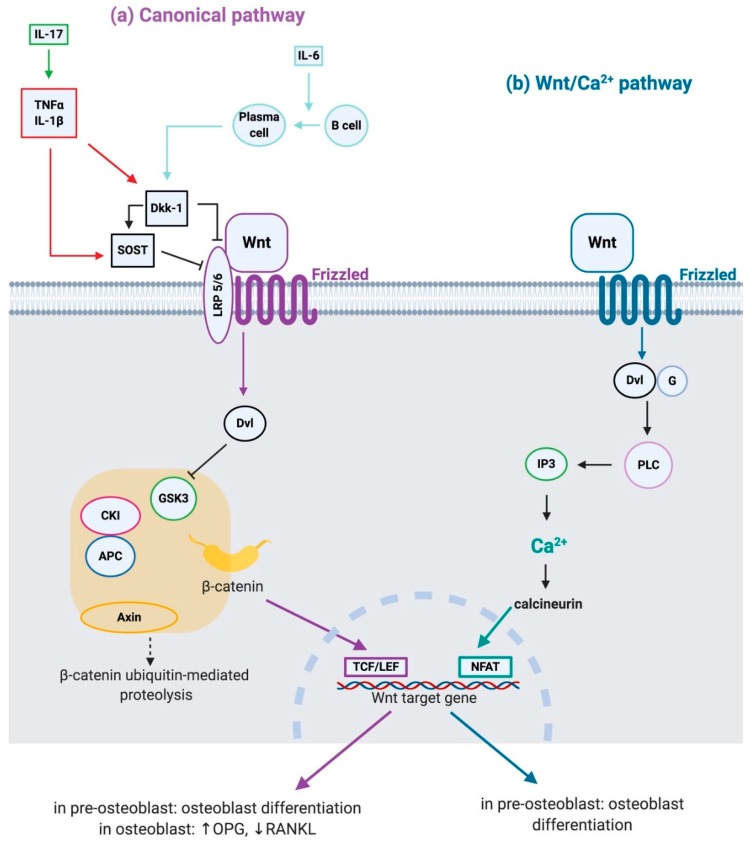
Wnt signaling pathways. (**a**) Canonical Wnt pathway: in the absence of Wnt ligands, β-catenin is degraded by a complex composed of glycogen synthase kinase-3β (GSK3), caseine kinase I (CKI), adenomatous polyposis coli (PCI) and Axin. Wnt proteins binding to their coreptor complex (Frizzled + low-density lipoprotein receptor-related protein (LRP) 5/6) leads to inhibition of GSK3 mediated by disheveled (Dvl) protein. In this condition, β-catenin translocates into the nucleus and together with T-cell factor (TCF)/lymphoid enhancer factor 1 (LEF) induces the expression of Wnt target genes, leading to osteoblast differentiation, up-regulation of osteoprotegerin (OPG) and down-regulation of Receptor activator of nuclear factor-kappa B ligand (RANKL). Dickkopf (Dkk) protein and sclerostin (SOST) inhibit this pathway by binding LRP5/6; moreover, Dkk-1 induces SOST. Pro-inflammatory cytokines tumor necrosis factor (TNF)α and interleukin (IL)-1β induce Dkk-1 and SOST; IL-17 down-regulates Wnt canonical pathway indirectly, enhancing the production of TNFα and IL-1β; furthermore, IL-6 induces differentiation of B cells into plasma cells which express Dkk-1. (**b**) Wnt/Ca^2+^ pathway: Wnt proteins binding to the Frizzled receptor lead to the activation of Dvl via activation of G-proteins (G). Dvl leads to cytoplasmic calcium (Ca^2+^) release from the endoplasmic reticulum via phospholipase C (PLC) and inositol 1,4,5-trisphosphate (IP3). Intracellular Ca^2+^ in turn activates calcineurin, which activates the nuclear factor of activated T-cells (NFAT), inducing the expression of Wnt target genes, leading to osteoblast differentiation.

The Wnt family in humans currently includes 19 different glycoproteins [2] that can trigger multiple signaling cascades: the “canonical pathway”, also known as the “Wnt/β-catenin pathway”, and several “noncanonical pathways” (Figure 1). To trigger the Wnt canonical pathway, Wnt ligands must bind their 7-pass transmembrane Frizzled receptors and the coreceptor low-density lipoprotein receptor-related protein (LRP) 5/6 [3]. In the absence of Wnt ligands, β-catenin is kept at a low level through the ubiquitin-proteasome system. It is located in an intracellular binding complex, composed of glycogen synthase kinase-3β (GSK3β), caseine kinase I (CKI), adenomatous polyposis coli (APC), and Axin [4]: GSK3β and CKI phosphorylate cytosolic β-catenin, Axin acts as a scaffold allowing the aforementioned phosphorylation and APC mediates phosphorylated β-catenin binding to the ubiquitin-mediated proteolysis pathway. The binding of the Wnt proteins to their coreceptor complex leads to the recruitment of the cytosolic disheveled proteins, which block the β-catenin degradation and allow its translocation into the nucleus, where it acts as a transcriptional regulator involved in the expression of several targeted genes [5].

The ‘‘noncanonical pathways’’ are numerous β-catenin independent signaling cascades, like planer cell polarity (PCP) and Wnt/calcium (Wnt/Ca^2+^) pathways. The PCP pathway is involved in cytoskeletal organization and coordinated polarization of cells within the plane of epithelial sheets [6]; the Wnt/Ca^2+^ pathway is involved in cancer, inflammation and neurodegeneration [7].

The Wnt signaling is regulated by different inhibitors, such as the secreted frizzled-related protein, the Wnt inhibitory factor 1, sclerostin and the Dickkopf (Dkk) family of secreted proteins. In particular, sclerostin is a protein encoded by the *SOST* gene primarily expressed by mature osteocytes, which decreases the life span of osteoblasts by stimulating their apoptosis; it inhibits Wnt signaling by binding the Wnt co-receptors LRP5/6 antagonizing downstream signaling; moreover, sclerostin also interacts with LRP4, a protein that specifically facilitates the aforementioned inhibitory action of sclerostin on Wnt/β-catenin signaling [8]. Dkk is a family of proteins comprising at least four different forms (Dkk-1 to Dkk-4); the best studied is Dkk-1, a powerful antagonist of canonical Wnt/β-catenin signaling: it binds with high affinity LRP5 and LRP6 and a single-pass transmembrane receptor, Kremen 1/2, which results in internalization of the complex and the consequent inhibition of Wnt signaling [9]. Moreover, Dkk-1 further inhibits Wnt signaling by inducing the other antagonist sclerostin [10]. Interestingly, it has been reported that 1,25(OH)_2_D_3_ plays a role in the regulation of the Wnt signaling pathway: indeed, it is able to induce the expression of LRP5 in osteoblasts targeting the *LRP5* gene [11].

The Wnt signaling is considered to be one of the main regulators of bone metabolism. It is involved in osteoblast differentiation from mesenchymal precursors and osteochondroprogenitor cells, in osteoblast regulation, proliferation and survival [1]. Moreover, it is also involved in osteoclastogenesis regulation: the canonical pathway leads to osteoprotegerin (OPG) expression, which acts as an inhibitor of the receptor activator of nuclear factor-kappa B (RANK)/RANK ligand (RANKL) signal, crucial to osteoclastogenesis [12]. Furthermore, Wnt inhibitor Dkk-1 seems to play a key role in osteoporosis [1,13,14]. In this regard, it is worth mentioning the emerging role of microRNAs (miRNAs) as regulators of the Wnt signaling. miRNAs are small noncoding RNA molecules which transcriptionally regulate gene expression by targeting mRNA with partially complementary sequences. Several miRNAs target key components of Wnt signaling cascade, leading to an attenuation or an enhancing of Wnt signals [15]. For example, the expression of miR-37c, miR-23a, miR-30e determines a decrease of Wnt signal and therefore a decreased osteoblast differentiation by targeting Wnt receptor or co-receptor (Wnt3, LRP5, LRP6). Conversely, miR-27a, miR-142, miR-29a, miR-218, miR-98, miR-335, miR-542 target Wnt inhibitors and negative regulators (such as APC, secreted frizzled-related proteins, Dkk-1, Kremen2, sclerostin), thus inducing Wnt signaling and osteogenic differentiation [15]. Moreover, a dysregulated expression of miRNAs targeting Wnt pathway components may lead to bone homeostasis impairment, such as osteoporosis. For example, an up-regulation of miR-320 could play a role in osteoporosis development through negatively regulating Wnt signal; on the contrary, miR-542 expression seems to prevent osteoporosis in mice models and it was down-regulated in postmenopausal osteoporotic patients [15]. Other non-coding RNAs, such as long non-coding RNAs and circular RNAs, act as regulators of Wnt pathway [16,17,18,19].

Inflammatory diseases are often characterized by bone metabolism impairment: local and systemic bone loss in RA, destructive and productive bone lesions in Spondyloarthritis (SpA). Interestingly, the Wnt signaling is believed to be implicated in the pathogenesis of many autoimmune diseases, including systemic lupus erythematosus, systemic sclerosis, rheumatoid arthritis (RA), ankylosing spondylitis (AS), psoriasis [1]. 

Autoimmune diseases are caused by a defective over activity of the immune system that leads the body to attack “self” components and damage its own tissues. The treatment for autoimmune diseases mainly focuses on relieving symptoms, given the incomplete understanding of their pathogenesis [20]. It has been shown that the Wnt signaling plays a regulatory role in the homeostasis of the immune system, as reviewed by Shi et al. [20]. It plays a key role in the maintenance, proliferation, differentiation, and self-renewal of hematopoietic stem cells, cells able to differentiate into hematopoietic progenitor cells, which can further differentiate into immune cells, such as T cells, B cells, NK cells, and macrophages. Moreover, several evidences support the role played by the Wnt pathway in immune cells differentiation and proliferation: the canonical pathway regulates T cell differentiation both in thymus and in peripheral lymphoid tissues, and its dysregulation could lead to autoimmunity or immune deficiency; the absence of Wnt-responsive transcription factors could lead to a defective development of T and B cells; the expression of a stable form of β-catenin in vitro enhances the survival of regulatory T (Treg) cells, therefore an activation of Wnt canonical pathway in inflammatory conditions could inhibit Treg cells function, thus triggering an immune response and the development of autoimmune responses. Furthermore, the Wnt signaling is also crucial for the differentiation of hematopoietic stem cells into normal functioning B cells; it is involved in the immune homeostasis through its activation in dendritic cells, regulating functions that contribute to the balance between tolerance and inflammation.

Interestingly, the Wnt signaling is down-regulated directly or indirectly by pro-inflammatory cytokines, such as tumor necrosis factor (TNF)α and interleukin (IL)-1 β (through the induction of Dkk-1 and sclerostin), IL-6, IL-17 [10,21,22]. Moreover, B cells differentiate into plasma cells which inhibit the Wnt signaling through the expression of Dkk-1 [23]. 

In the last few decades, there has been a revolution in the treatment of chronic inflammatory rheumatic diseases. Biological agents able to inhibit molecular targets directly involved in the pathogenesis of these diseases have been developed, dramatically improving the quality of life of patients suffering from inflammatory joint diseases, particularly RA and Spondyloarthritis (SpA). Anti-TNFα agents have been approved for the treatment of various inflammatory diseases, and have proven effective in RA, psoriatic arthritis (PsA), AS; they represent the most used biologic drugs since 2000 and are usually well tolerated. Anakinra is a human IL-1 receptor antagonist that acts by competitively inhibiting the binding of IL-1 with the IL-1 type 1 receptor; currently approved for the treatment of RA, cryopyrin-associated periodic syndromes, and Still’s disease [24]. Tocilizumab is an IL-6 receptor antagonist approved for the treatment of adults with moderate to severe active RA. Its safety and short- and long-term efficacy have been demonstrated, in terms of clinical and radiographic outcomes and health-related quality of life [25]. Secukinumab is a monoclonal antibody which selectively binds to and neutralizes IL-17A, effective in the treatment of psoriasis and PsA [26]. 

The aim of this review is to focus on the relation between the Wnt signaling and the biological therapy for RA and SpA (Table 1). A literature search for reviews and studies in PubMed, Google Scholar and Scopus databases was performed. The search was limited to the last ten years and only articles with abstract in English language were considered. The keywords used were “wnt signaling”, “wnt rheumatoid arthritis”, “wnt spondyloarthritis”, “wnt psoriatic arthritis”, “wnt ankylosing spondylitis”, “dkk rheumatoid arthritis”, “dkk spondyloarthritis”, “wnt osteoporosis”, “wnt biological therapy”, “abatacept wnt”, “tnf wnt”. We scanned the reference list of the selected articles to identify other relevant papers.

## 2. Wnt Signaling in RA

RA is a chronic autoimmune inflammatory disease, that primarily attacks synovial joints. It is characterized by symmetric polyarticular inflammation, most commonly involving the small joints of hands and feet, which can lead to progressive joint damage and resulting functional disability with a decreased quality of life. 

Several studies support the hypothesis of a potential involvement of Wnt signaling in the etiology of RA, in particular Wnt7b [20]: higher levels of Wnt ligands, Frizzled receptors, and Wnt inducible signaling pathway proteins were observed in the synovium of RA patients, also as pro-inflammatory cytokines TNFα, IL-1β and IL-6. Moreover, it has been demonstrated that Wnt inhibitor Dkk-1 promotes synovial angiogenesis, a critical process in the pathogenesis of RA [35]: vascular proliferation occurs during pannus formation in the affected joints [36,37], during which the synovium becomes locally invasive at the interface with cartilage and bone. The pannus plays a key role in the joint damage observed in RA [37]. Furthermore, also miRNAs have been implicated in the pathogenesis of RA by targeting Wnt signaling pathways. Indeed, a downregulated expression of miR-152 was found in arthritic rat model, whereas upregulation of this miRNA in fibroblast-like synoviocytes indirectly upregulated the expression of secreted frizzled-related protein 4, a Wnt negative regulator, thus leading to canonical Wnt pathway inhibition and to a significant decrease of fibroblast-like synoviocytes proliferation [38]. Consistently, miR-375 was also downregulated in fibroblast-like synoviocytes of arthritic rat model, and its upregulation inhibited the canonical Wnt pathway by targeting Frizzled 8. Interestingly, increased miR-375 also inhibited the pathogenesis of arthritis in the rat model, as indicated by decreases in disease markers, showing a role played by this miRNA in the pathogenesis of the disease through the canonical Wnt signaling pathway [39]. In addition, in synovium from RA patients, miR-663 was found upregulated, whereas APC expression was decreased. It was therefore revealed that this miRNA could active the canonical Wnt pathway by targeting APC [40].

RA is characterized by bone involvement represented by generalized osteoporosis and localized bone loss, which includes erosions and iuxta-articular osteopenia of affected joints [41].

Both bone erosions and systemic bone loss are due to an imbalance of the osteoblast–osteoclast axis. The chronic inflammatory state typical of RA leads to bone loss: pro-inflammatory cytokines TNFα, IL-1β and IL-6 upregulate the RANK/RANKL pathway, leading to increased osteoclast activity, prolonged osteoclast lifespan and bone resorption [42]. OPG acts as a competitive inhibitor of RANKL, reducing osteoclastogenesis and bone resorption. Moreover, several evidences suggest a connection between systemic osteoporosis in RA patients and low 25(OH) vitamin D levels. In numerous studies conducted on RA patients, low levels of 25(OH) vitamin D showed a significant association with disease activity and bone loss. As a matter of fact, vitamin D acts as a wide spectrum immunomodulator, with effect on several cells of the immune system (B and T cells, monocytes/macrophages), promoting an anti-inflammatory immune status [42].

The Wnt signaling plays a central role in bone development and homeostasis: its canonical pathway leads to osteoblast commitment, proliferation, and differentiation, enhances osteoblast and osteocyte survival; furthermore, it has been suggested that *OPG* gene expression is regulated by the Wnt/β-catenin signaling pathway [12]. Indeed, animal studies show that Wnt signaling inhibitor Dkk-1 is able to downregulate OPG and upregulate RANKL expression [43]. In addition, Wnt canonical pathway is downregulated in systemic osteoporosis: several studies found monogenic causes of osteoporosis [44], like mutations in genes encoding for LRP5 [45,46] and different Wnt proteins [47,48,49,50,51]. Moreover, the Wnt inhibitors Dkk-1 and sclerostin play a key role in systemic bone loss. Dkk-1 levels are higher in postmenopausal osteoporosis patients than in age-matched controls [13], and in osteoporosis patients higher Dkk-1 serum levels highly correlated with bone mass variables with inverse associations found between serum Dkk-1 expression and lumbar and femur T-score [52]; Dkk-1 expression in osteoblastic cultures from osteoporotic subjects is higher than in control cells [14]. Further studies suggest that Dkk-1 is a key inhibitor of systemic bone formation, like increased bone mass and osteoblast activity in Dkk-1 deficient mice [27,53] and osteopenia in mice over-expressing Dkk-1 [30]; in naïve normal growing female mice, human monoclonal anti-Dkk-1 antibodies are able to significantly improve both trabecular and cortical bone mineral densities [28].

Overexpression of *SOST* gene in mice leads to low bone mass as a result of reduction in osteoblast activity [29]; on the contrary, loss-of-function mutations of *SOST* gene lead to van Buchem disease and sclerosteosis, diseases distinguished by osteoblast hyperactivity and consequent increased bone density [54]. Interestingly, dual inhibition of sclerostin and Dkk-1 with a bispecific antibody resulted in a greater increased bone formation when compared to neutralization of each of the Wnt antagonists alone. These evidences further prove the synergical action of sclerostin and Dkk-1 in inhibiting the Wnt pathway [8].

Wnt signaling is involved in the bone alterations typical of RA: systemic and localized bone loss. In this regard, in the latest years, Wnt inhibitors Dkk-1 and sclerostin have gained more importance in understanding the physiopathological processes underlying RA. In a recent study by Singh et al., sclerostin serum levels were significantly higher in RA patients than in controls, and showed significant correlation with disease activity scores and inflammation markers, but not with bone destruction [55]. In animal models, Dkk-1 blockade in TNF-transgenic mice prevented the development of bone erosions [10]. Moreover, treatment with sclerostin antibodies in human TNF transgenic mice with late stage inflammatory arthritis, lead to a great improvement in bone complications: in this study, the treatment reversed systemic and periarticular osteopenia, improved articular cartilage damage, blocked progression of bone erosion and, when combined with TNF inhibitors, it was also able to reverse and repair articular cartilage damage and cortical bone erosions [31].

Interestingly, different studies showed that serum level of Dkk-1 is higher in patients with RA than in controls, and correlates with disease activity, elevated acute-phase reactants and more severe bone destruction in patients with RA [35,55,56,57]. Dkk-1 may be a biomarker of structural damage and a predictor of structural progression; therefore, it could represent a therapeutic target in RA [56]. In this regard, treatment with anti-Dkk-1 antibodies seems promising: in mouse models of RA, anti-Dkk-1 antibodies were able to block inflammatory bone erosions, as demonstrated by radiographic and histopathological examinations, due to a decreased osteoclast formation in the affected joints [22]. More evidences supporting the role of Wnt in bone homeostasis in RA come from studies focused on abatacept (CTLA-4Ig). Abatacept inhibits TNF-mediated osteoclastogenenis in vitro without T cells and inhibits inflammatory bone erosion in vivo in an animal model of RA [58]. CTLA-4Ig is a recombinant fusion protein based on CTLA-4, a protein physiologically produced by activated T cells that functions as a negative regulator of T cell-mediated immune responses. Indeed, full T cell activation requires two signals: the initial recognition of a specific antigen by a T cell receptor, after which T cells are unresponsive to further antigenic presentations (anergy); secondly, a co-stimulatory signal, such as the binding of CD80 or CD86 on the CD28 receptor on T cells. Abatacept and CTL4-a inhibit T cell activation by binding to CD80 and CD86 [59,60].

Roser-Page et al investigated the effect of abatacept on bone turnover in mice and found that its anabolic activity is linked to Wnt signaling [59,61]. The authors reported that CTLA-4Ig administration in healthy mice induced bone gain due to an increased osteoblast bone formation and they also found increased expression of Wnt ligand Wnt-10b in purified T cells from the animals. The authors therefore hypothesized that abatacept induces Wnt-10b in anergized T cells, therefore promoting bone formation [59]. These findings were later confirmed by another study from the same research group conducted on mouse genetic models of Wnt-10b and T cell deficiency [8]. The data collected supported the hypothesis that the anabolic activity of abatacept in vivo is indeed mediated by a mechanism involving T cells and Wnt-10b: in the absence of either T cells or Wnt-10b, CTLA-4Ig did not lead to bone formation, on the contrary its effect was a significant loss of bone mass. Moreover, the authors also reported an increased expression of sclerostin following abatacept administration in healthy mice. Therefore, the authors hypothesized that in basal conditions, abatacept-induced Wnt-10b expression from T cells compensates for production of sclerostin and CTLA-4Ig promotes bone formation; however, in the absence of Wnt-10b, the net effect of abatacept is increased sclerostin production and consequent suppression of bone formation. These findings suggest caution in the treatment of RA immunocompromised patients, in whom abatacept administration may lead to unpredictable outcomes on bone formation [61].

These findings indicate that bone complications typical of RA might be the result of both enhanced bone resorption and impaired bone formation, in consequence of increased TNFα-driven osteoclast activity and overproduction of Wnt inhibitor Dkk-1, both locally (erosions) and systemically (RA-associated osteoporosis) [62]. 

Biological therapy is able to slow down bone destruction and inhibit radiological progression in RA [63]. Several studies show how this effect may be connected with the Wnt signaling. The studies evaluating the effect of biological therapy on Wnt pathway in RA were mainly focused on TNFα inhibitors, IL-1 receptor antagonist (IL-1Ra) anakinra and anti-IL-6 monoclonal antibody tocilizumab.

Most of the studies were conducted with TNFα inhibitors, showing a decrease in serum concentration of Dkk-1 in RA patients undergoing this biological treatment [34,35,43,62]. In particular, Adami et al. found a reduction in Dkk-1 serum levels in RA patients after 6 months of anti-TNFα treatment (most patients were treated with adalimumab and certolizumab, less with etanercept, infliximab and golimumab); they did not observe any changes in sclerostin serum levels, therefore suggesting that the changes of Dkk-1 were not associated with a feedback with sclerostin [62]. Conversely, Fassio et al. recently found a rapid decline in both Dkk-1 and sclerostin serum levels in RA patients after 2 months of treatment with TNFα inhibitor certolizumab-pegol, accompanied by dramatic changes in bone turnover [43]. The rapidity of the reduction in serum Dkk-1 levels observed after initiation of the treatment further proves the TNFα-dependent induction of Dkk-1 in vivo, already demonstrated in vitro [22], as also suggested by Daoussis et al [34]. Wang et al. found that the serum Dkk-1 concentration was significantly increased in RA patients than in healthy controls and patients affected by other rheumatic diseases; moreover, increasing Dkk-1 levels were associated with bone erosion and correlated with levels of inflammatory markers like serum C-reactive protein levels and erythrocyte sedimentation rates. Furthermore, in their study, treatment with anti-TNFα agent infliximab and anti-IL-1 anakinra induced a significant reduction of circulating levels of Dkk-1 [35]. Therefore, Dkk-1 may serve as a new clinical indicator for RA: a biomarker of disease activity and bone erosion, as well as a marker of response to biological therapy with TNFα blockers and IL-1Ra. These evidences suggest the strong connection between the dysregulation of the Wnt signaling pathway, TNFα-dependent inflammation and bone metabolism alterations in RA. 

The key role of IL-6 in the pathogenesis of bone changes in RA through the regulation of the Wnt pathway has been suggested by studies evaluating the levels of Dkk-1 in RA patients after treatment with anti-IL-6 monoclonal antibody tocilizumab. In the 1-year prospective open study conducted by Briot et al. [64], a significant decrease in serum level of Dkk-1 was observed following tocilizumab administration, confirming the results from a previous study by Terpos et al. [65]. It has been suggested that tocilizumab does not affect the whole osteocyte functions since no significant change was observed in sclerostin serum levels [64]. These studies suggest a critical role of IL-6 in bone homeostasis in RA and how its dysregulation may be connected with the bone complications of this disease. Indeed, several in vitro and animal studies support this theory [65]: increased osteoclastogenesis and reduced osteoblast activity in IL-6 transgenic mice; decreased levels of RANKL produced by T-lymphocytes in IL-6-deficient mice; furthermore, blockade of IL-6 receptor through neutralizing antibodies blocks TNF- and RANKL-mediated osteoclastogenesis in vitro and in vivo as shown by Axmann et al. [32]. Thus, anti-IL-6 therapy may have a direct effect on bone metabolism impairment in RA patients.

## 3. Wnt Signaling in SpA

The term SpA refers to a group of inflammatory rheumatic diseases that share genetic background, pathophysiological mechanisms, and clinical features. Depending on the leading manifestation, SpAs can be classified as axial (mainly involving the axial skeleton, i.e. sacroiliac joints and spine) or peripheral (distinguished by arthritis, enthesitis or dactylitis). The latter includes reactive arthritis, arthritis associated with inflammatory bowel disease, PsA and so-called undifferentiated peripheral SpA [66]. Unlike RA bone involvement, SpA is characterized by the co-existence of both destructive and productive bone lesions, which suggests an alteration of a bone remodelling in the affected joints [33,67,68].

In particular, in PsA bone erosions are associated with new bone formation, typically at the edges of the cartilage joint at the insertion of the enthesis [69]. IL-17 is an important cytokine involved in the pathogenesis of bone lesions of PsA, via the inhibition of the Wnt signaling [70]. In a recent study conducted by Fassio et al. [26] serum levels of Dkk-1 in PsA patients were found lower than in healthy subjects and significantly increased after treatment with monoclonal anti-IL-17A antibody secukinumab. The aforementioned treatment appeared to restore normal Dkk-1 serum levels in these patients, resulting in the loss of the significant gap vs the healthy controls after six months of therapy. These findings suggest a drug-induced inhibition of local bone over-proliferation, typical of the bone lesions in PsA.

### Axial SpA (axSpA)

Pathologically, axSpA is characterized by a three-stage process, resulting in a paradoxical bone destruction and formation. It has been postulated that the initial inflammation (in which TNFα is the principal cytokine involved [71]) causes erosions in cartilage and bone; these lesions are then filled in by fibrous tissue, that is later ossified leading to abnormal bony outgrowth (syndesmophytes, bone bridges and complete ankyloses) [9,68,72]. The Wnt signaling and its inhibitors, such as Dkk-1 and sclerostin, might play a role in the third phase of axSpA, due to their role in bone homeostasis [9,73]. 

In AS the primary site of inflammation is located at the enthesis or subchondral bone marrow, with bone marrow edema, lymphocytic infiltrates, increased osteoclast density, and increased microvessel density as typical findings in acute inflammation [35]. TNFα is a primary cytokine in axSpa [73], not only for its pro-inflammatory effect [71,74], but also as a stimulator of osteoclastogenesis [67] and for its effect on the Wnt signaling (enhancing the expression of Wnt inhibitors, Dkk-1 and sclerostin [10,21,23,63,75]).

Treatment with TNFα inhibitors has greatly improved the clinical outcome of axSpA patients, reducing the signs and symptoms improving the health-related quality of life. However, it is still unclear whether they slow the structural damage and radiographic progression or not [63,67,68]. In this context, several studies focused on the role of Dkk-1 in axSpA, in relation to bone formation and treatment with TNFα inhibitors [33,34,76,77,78].

Studies have shown that Dkk-1 is involved in the pathogenesis of structural damage and in molecular mechanisms of syndesmophyte formation and new bone formation in SpA, but most data are conflicting [33,34]. Various studies showed that Dkk-1 circulating levels respond in a different way to TNFα blockade in patients with AS and in patients with RA. It has been shown that serum levels of Dkk-1 were significantly higher in patients with AS compared to patients with RA and healthy controls and were further increased in AS patient receiving anti-TNFα treatment [34]; the authors hypothesized that the higher levels of Dkk-1 may reflect a counter-balancing mechanism to attenuate Wnt signaling, which is turned on after resolution of inflammation following the treatment. Conversely, another study revealed that circulating levels of Dkk-1 were lower in patient with AS compared to healthy controls and did not change after anti-TNFα treatment [33]. It could be therefore hypothesized that in AS the circulating levels of Dkk-1 are unable to suppress Wnt-mediated bone formation, and thus the inability of TNFα to induce Dkk-1 in SpA may be one explanation why anti-TNFα therapy might not block new bone formation [63]. 

Levels of functional Dkk-1 are increased in AS patients with no syndesmophyte growth compared to patients with syndesmophyte growth, suggesting that low levels of serum DKK-1 are necessary to develop syndesmophytes. Therefore, inhibition of Wnt signaling could protect from the development of syndesmophytes through an inhibition of bone formation processes [76]. In an animal model of transgenic mice overexpressing TNF, which develops bilateral sacroiliitis, Dkk-1 blockade promotes the expression of type X collagen and the formation of hypertrophic chondrocytes, promoting the ankylosis of sacroiliac joints, without affecting inflammatory processes [77]. Nevertheless, a prospective study on SpA patients showed that treatment with TNFα blockers induced a significant decrease of circulating levels of Dkk-1, which correlated with the reduction of MRI bone marrow edema of sacroiliac joint and spine, suggesting that the reduction of Dkk-1 by anti-TNFα agents could be related to the inhibitory effects of these drugs on new bone formation in SpA [78].

The hypothesis that the Wnt pathway plays an important role in the pathogenesis of AS is supported by studies that focused on another Wnt signaling inhibitor, sclerostin. It was observed that sclerostin expression was nearly absent in osteocytes in the periarticular bone of AS patients, suggesting a specific alteration of osteocyte function in this disease [79]. Moreover, serum sclerostin levels were found lower in AS patients than in healthy controls [79,80]. Interestingly, radiographic progression was associated with significantly lower sclerostin levels, suggesting that a low sclerostin level in AS could increase susceptibility for syndesmophyte formation [79]. Furthermore, although increasing after anti-TNFα treatment, sclerostin levels were still lower than in healthy subjects after 12 months of therapy [80]. Moreover, since AS patients with higher baseline sclerostin levels showed a rapid reduction of inflammatory parameters following the treatment and a concomitantly gradual increase in this Wnt inhibitor, the authors hypothesized that low sclerostin serum levels could be a possible marker of persistent inflammation in AS patients under anti-TNF therapy. 

These data confirm the potential role of Wnt signaling in the pathogenesis of structural bone changes in SpA. Several physiopathogenetic hypothesis have been proposed to explain the conflicting available data on Wnt involvement in bone changes observed in SpA patients. There is a difference between the total levels of Dkk-1 and receptor-bound and thus functional Dkk-1, showing a minor capacity of Dkk-1 in axSpA patients to bind the LRP coreceptor [22,34,76]. Another explanation could be that Dkk-1 serum levels in patients with axSpA could be related to disease duration [72], as Dkk-1 serum levels were found higher in patients with early axSpA compared with patients with established disease. 

This explanation may also clarify the link between TNFα and Dkk-1, and could contribute to explain the relationship between inflammation and osteoproliferation. The lower levels of Dkk-1 in patients with established axSpA may explain why TNFα blockers in this stage of the disease did not lead to a change in Dkk-1 levels in the study conducted by Kwon et al. [33] and is not able to avoid the development of syndesmophytes [68]. On the contrary, in patients with early axSpA, anti-TNFα treatment could lead to a decrease in Dkk-1 serum levels [75,78] and also to a better radiographic outcome, as shown by a longitudinal cohort study conducted by Haroon et al. [81]. In this study, radiographic progression was slower in AS patients who underwent TNFα-blocker therapy earlier in the course of disease than in patients in whom treatment was delayed. Indeed, in the early stages of the disease, inflammation and osteoproliferation may be coupled. Considering the three-stage pathological process of axSpA (inflammation, erosion, osteoproliferation), targeting the inflammation with TNFα inhibitors in patients at an early stage of the disease, may avoid the development of the next stages and thus the new bone formation processes. In other terms, if early inflammatory lesions resolve without undergoing chronic changes, the sequelae of new bone formation following inflammation might be halted [68,72]. Therefore, anti-TNFα treatment in the early stages of the disease may prevent development of osteogenesis, while at later stages osteogenesis and inflammation appear to be two separate therapeutic targets [63,68,75].

The clinical application of this theory is to establish the therapeutic time window to avoid the new bone formation process and improving the radiographic outcome through TNFα inhibitors, before the disconnection between inflammation and osteoproliferation [75]. This window might be characterized by higher serum levels of Dkk-1 in axSpA patients than in controls.

## 4. Conclusions

The Wnt signaling is involved in pathological aspects of rheumatic diseases, with several evidences supporting its role especially in bone complications, bone loss and new bone formation. 

Higher serum levels of Wnt inhibitor Dkk-1 found in RA patients than in controls and its decrease after biological treatment (TNFα inhibitors, IL-1Ra, anti-IL-6 antibody), suggest the strong connection between the dysregulation of Wnt signaling leading to bone loss—both locally and systemically—in RA and the pro-inflammatory state typical of the disease. Interestingly, Dkk-1 concentration correlates with disease activity, elevated acute-phase reactants and more severe bone damage, suggesting its role as a possible biomarker of structural damage and new therapeutic target in RA. Moreover, in RA patients, higher levels of another Wnt inhibitors, sclerostin, correlates with disease activity scores and inflammation markers; in addition, in RA mice models anti-sclerostin antibodies arrested the bone damage progression and in combination with anti-TNF led to regression of cortical bone erosions.

In axSpA, the role of Wnt signaling has not yet been fully understood. There is evidence of a critical time window in the early stages of the disease when treatment with TNFα inhibitors might lead to two otherwise separate therapeutic targets: reducing signs and symptoms, and slowing the structural damage and radiographic progression. Further exploration of Dkk-1’s role and the connection between bone formation and inflammatory response is needed in order to achieve a better knowing of the biologic background of the disease; this would subsequently lead to better management of current therapeutic options.

## Figures and Tables

**Table 1:** Wnt signaling and biological therapy in arthritis.

Study	Disease	Patients	Observation Time	Treatment	Effect on Wnt Signaling	Reference
Adami et al. 2016	RA	54	6 months	TNFi (adalimumab, certolizumab, etanercept, infliximab, golimumab)	↓ Dkk-1 ↔ sclerostin	[27]
Fassio et al. 2019	RA	17	2 months	TNFi (certolizumab)	↓ Dkk-1 ↓ sclerostin	[25]
Wang et al. 2011	RA	100	6 months	TNFi (infliximab); IL-1Ra (anakinra)	↓ Dkk-1	[21]
Briot et al. 2015	RA	103	1 year	Anti-IL-6 (tocilizumab)	↓ Dkk-1 ↔ sclerostin	[28]
Terpos et al. 2011	RA	22	8 weeks	Anti-IL-6 (tocilizumab)	↓ Dkk-1	[29]
Fassio et al. 2019	PsA	28	6 months	Anti-IL-17 (secukinumab)	↑ Dkk-1	[20]
Daoussis et al. 2010	AS	45	3 months	TNFi	↑ Dkk-1	[30]
Kwon et al. 2012	AS	56	3 months	TNFi (etanercept, adalimumab, infliximab)	↔ Dkk-1	[31]
Zhao et al. 2019	SpA	30	6 months	TNFi	↓ Dkk-1	[32]
Saad et al. 2012	AS	30	1 year	TNFi (infliximab, adalimumab, etanercept)	↑ sclerostin	[33]
Korkosz et al. 2014	AS	40	6 months	TNFi (etanercept, adalimumab)	↓ Dkk-1	[34]

RA: rheumatoid arthritis; PsA: psoriatic arthritis; AS: ankylosing spondylitis; SpA: spondyloarthritis; TNFi: tumour necrosis factor inhibitor; Il-1Ra: IL-1 receptor antagonist; ↓: decreased; ↔: unchanged; ↑: increased.

## Abbreviations

AS	Ankylosing spondylitis
axSpA	Axial spondyloarthritis
CKI	Caseine kinase I
DKK	Dickkopf
GSK3β	Glycogen synthase kinase-3β
IL	Interleukin
IL-1Ra	IL-1 receptor antagonist
LRP	Low-density lipoprotein receptor-related protein
OPG	Osteoprotegerin
PCK/Ca^2+^	Protein kinase C/calcium
PCP	Planer cell polarity
PsA	Psoriatic arthritis
RA	Rheumatoid arthritis
RANK	Receptor activator of nuclear factor-kappa B
RANKL	RANK ligand
SpA	Spondyloarthritis
TNF	Tumor necrosis factor
Treg	Regulatory T
